# Visual and Super-Sensitive Detection of Maize Chlorotic Mottle Virus by Dot-ELISA and Au Nanoparticle-Based Immunochromatographic Test Strip

**DOI:** 10.3390/v15071607

**Published:** 2023-07-22

**Authors:** Cui Zhang, Mengmeng Guo, Jinxi Dong, Li Liu, Xueping Zhou, Jianxiang Wu

**Affiliations:** 1 State Key Laboratory of Rice Biology, Key Laboratory of Biology of Crop Pathogens and Insects of Zhejiang Province, Institute of Biotechnology, Zhejiang University, Hangzhou 310058, China; 12216095@zju.edu.cn (C.Z.); 22016081@zju.edu.cn (M.G.); 22016215@zju.edu.cn (J.D.); 2 Hainan Institute, Zhejiang University, Sanya 572025, China; 3 The Department of Applied Engineering, Zhejiang Economic and Trade Polytechnic, Hangzhou 310018, China; 4 State Key Laboratory for Biology of Plant Diseases and Insect Pests, Institute of Plant Protection, Chinese Academy of Agricultural Sciences, Beijing 100193, China

**Keywords:** maize chlorotic mottle virus, monoclonal antibody, Dot-ELISA, immunochromatographic test strip, RT-PCR

## Abstract

Maize chlorotic mottle virus (MCMV) is the only species in the *Mahromovirus* genus and is often co-infected with one or several viruses of the *Potyvirus* genus, posing a great threat to the global maize industry. Effective viral integrated management measures are dependent on the timely and proper detection of the causal agent of the disease. In this work, six super-sensitive and specific monoclonal antibodies (mAbs) against MCMV were first prepared using purified MCMV virions as the immunogen. Then, the Dot enzyme-linked immunosorbent assay (Dot-ELISA) was established based on the obtained mAbs, and it can detect MCMV in infected maize leaf crude extracts diluted up to 1:10,240-fold (*w*/*v*, g/mL). Furthermore, a rapid and user-friendly Au nanoparticle-based immunochromatographic test strip (AuNP-ICTS) based on paired mAbs 7B12 and 17C4 was created for monitoring MCMV in point-of-care tests, and it can detect the virus in a 25,600-fold dilution (*w*/*v*, g/mL) of MCMV-infected maize leaf crude extracts. The whole test process for ICTS was completed in 10 min. Compared with conventional reverse transcription-polymerase chain reaction (RT-PCR), the detection endpoint of both serological methods is higher than that of RT-PCR, especially the Dot-ELISA, which is 12.1 times more sensitive than that of RT-PCR. In addition, the detection results of 20 blinded maize samples by the two serological assays were consistent with those of RT-PCR. Therefore, the newly created Dot-ELISA and AuNP-ICTS exhibit favorable application potential for the detection of MCMV in plant samples.

## 1. Introduction

Maize chlorotic mottle virus (MCMV), first discovered in Peru in 1971 [1], is the only identified member of the *Machlomovirus* genus from the *Tombusviridae* family [2]. MCMV naturally infects maize, sugarcane, and other monocotyledon plants by seeds, mechanical means, and insects, causing varying degrees of symptoms, from mild mottling to severe mosaic and developmental delay [3]. While MCMV often co-infects with one or several viruses from the *Potyvirus* genus, such as maize dwarf mosaic virus (MDMV) [4], johnsongrass mosaic virus (JGMV) [5], sugarcane mosaic virus (SCMV) [6,7,8], and wheat streak mosaic virus (WSMV) [9], causing maize lethal necrosis disease (MLND) [10], which has posed a great threat to the global maize industry. MLND-diseased Maize plants usually exhibit yellow streaks, chlorotic mottling, and leaf necrosis, leading to a considerable yield loss [11,12]. In 2010, our group first reported MLND caused by the co-infection of MCMV and SCMV in China [13]. Wei et al. evaluated the potential threat of MCMV to China’s maize industry using @RISK software based on stochastic models and found that without prevention and control, the potential economic loss of MCMV could reach 3.3–5.1 billion in China [14]. Timely and proper detection and diagnosis play a key role in controlling plant viral diseases.

MCMV is a single-stranded, positive-sense RNA virus with a length of 4437 nucleotides lacking a poly(A) tail at the 3′ terminus and a genome-linked protein (Vpg) at the 5′ terminus [15]. During infection, a single, 3′ co-terminal 1.3 kb sub-genomic RNA was produced that encodes a coat protein (CP) of 25 KDa. The genome contains four long open reading frames (ORFs) encoding a total of seven proteins, namely P32, P50, P111, P31, P7a, P7b, and CP [2]. P32, unique to MCMV, is the first protein encoded by the 5′ terminal ORF1 of the MCMV genome with a molecular weight of 32 KDa, which is not essential for viral replication or movement. P50 and its stop codon read-through protein, p111, are the proteins required for replication in maize protoplasts and are associated with highly conserved RNA-dependent RNA polymerases. The protein p31 is the major pathogenic determinant of MCMV and is essential for viral accumulation and symptom development [16]. Movement proteins P7a and P7b, encoded by subgenomic RNA1, are required for cell-to-cell movement. Under transmission electron microscopy (TEM), MCMV virions are spherical particles with a diameter of about 30 nm without an envelope.

So far, a variety of methods have been developed for determination of MCMV infection, including enzyme-linked immunosorbent assay (ELISA)-based serological methods such as triple antibody sandwich enzyme-linked immunosorbent assay (TAS-ELISA) [17], dot-immunobinding assay (DIBA) [17], and double antibody sandwich-ELISA (DAS-ELISA) [7,18], as well as polymerase chain reaction (PCR)-based molecular biological technologies such as reverse transcriptional (RT)-PCR [7,19,20,21], real-time TaqMan RT-PCR [22], RT-recombinase polymerase amplification (RPA) assay [23], reverse transcriptional loop-mediated isothermal amplification, (RT-LAMP) [24,25], next-generation sequencing [26], and CRISPR/Cas12a-based detection method [27]. Molecular biological technologies are highly specific and sensitive; however, they are unsuitable for on-site rapid detection of MCMV in field surveys and for fast inspection and quarantine at ports because of their tedious conducting procedures, high cost, and long detection time. Compared to Molecular biological methods, serological immunoassays are often used to monitor large numbers of samples owing to their low cost, high specificity, and high throughput. Nevertheless, ELISA-based methods often require multiple washing and reaction steps and a relatively long test time, which greatly limits their application in point-of-care tests [28,29,30]. Therefore, a rapid, simple, sensitive, and user-friendly test tool for the detection of MCMV is crucial for effective viral integrated management measures. Immunochromatographic test strips (ICTS), also known as lateral flow immunoassays, are widely applied in various areas ranging from diagnostics to environmental and safety analyses due to their visual readout, rapidity, low cost, and lack of need for any instruments [31,32,33,34]. Currently, the most widely used ICTS is the Au nanoparticle (AuNP)-based ICTS, which uses AuNPs as signal reporters and carriers. In this study, we describe the use of hybridoma technology to obtain monoclonal antibodies (mAbs) against MCMV to develop accurate diagnostic tools such as the Dot enzyme-linked immunosorbent assay (Dot-ELISA) and AuNP-ICTS.

## 2. Materials and Methods

### 2.1. Virus Sources, Virion Purification

Uninfected maize plants are planted in our laboratory glasshouse. Maize leaves infected with MCMV were collected from maize fields in Yunnan Province, China, intercepted at customs ports, and kept in our laboratory refrigerators. After being ground in the inoculation buffer (0.1 M pH 7.2 phospholipid buffer), the resulting homogenate was wiped onto the leaves of healthy maize seedlings planted in our laboratory glasshouse. RT-PCR and DNA sequencing were used to determine whether MCMV was successfully inoculated 2 weeks after inoculation. MCMV virions are finally extracted from the inoculated and diseased maize leaves by the differential centrifugation method described previously [35].

### 2.2. Production and Characterization of Anti-MCMV mAbs

To produce anti-MCMV mAbs, five eight-week-old BALB/c female mice were immunized three times with the purified MCMV virions as described previously [36]. Briefly, the purified MCMV (80 µg/mouse) was intraperitoneally injected into mice with Freund’s complete adjuvant (Sigma-Aldrich, Saint Louis, MO, USA) for the primary immunization and with Freund’s incomplete adjuvant for the boost immunization at 21-day intervals. Three days before cell fusion, the mice were immunized intraperitoneally with 100 μg of purified MCMV virions in phosphate buffered saline (PBS, 0.1 M, pH 7.4). Then, splenocytes were isolated from immunized mice and fused with myeloma cells Sp2/0 via the standard polyethylene glycol (MW3350, Sigma-Aldrich) protocol. The fused cells were cultured in the supplementary HAT selection medium, i.e., RPMI-1640 medium (Gibco, Amarillo, TX, USA), supplemented with 100 mol/L hypoxanthine, 0.4 mol/L aminopterin, 16 mol/L thymidine, 10% fetal bovine serum (Hangzhou Jiangbin Biotechnology Co., Ltd., Hangzhou, China), and 1% penicillin-streptomycin (Gibco, Grand Island, NY, USA). The supernatant from individual hybridoma cultures was screened by indirect ELISA for anti-MCMV antibody production. The hybridomas secreting antibodies to MCMV were cell-cloned using the limiting dilution method [37]. The monoclonal hybridoma lines were injected intraperitoneally into syngeneic mice to prepare ascites containing MAbs. Subsequently, the mAbs in ascitic fluids were purified by saturated ammonium sulfate precipitation and stored at −80 °C for further use. Finally, the selected mAbs were identified by Western blot and Dot-ELISA assays [38,39], and the isotypes of the mAbs were determined using a mouse mAb isotyping kit (Sigma-Aldrich).

### 2.3. Pretreatment of Maize Leaf Samples

For analysis, 0.1 g of maize leaf samples were first ground in 2 mL of 0.01 M PBS, and the homogenate was centrifuged at 4 °C for 5 min at 5000 rpm, or without centrifugal procedure. Subsequently, the resulting supernatant or homogenate was used as the plant tissue crude extract, transferred into a new tube, and subjected to a 2-fold gradient dilution in PBS for analyzing the sensitivity of Dot-ELISA or AuNP-ICTS. In Dot-ELISA, 2 µL of maize leaf crude extracts (per sample) were dropped on the nitrocellulose membranes, and in the AuNP-ICTS test, 60 µL~100 µL of maize leaf crude extracts were dropped into the sample pad of AuNP-ICTS.

### 2.4. Development of Dot-ELISA for the Detection of MCMV

Dot-ELISA is a modified ELISA technique based on sample-dotted nitrocellulose membranes. The Dot-ELISA procedure was carried out as described previously [40]. Briefly, 2 µL of maize leaf crude extract samples were pipetted evenly onto the nitrocellulose membranes (GE Healthcare, Bucks, UK) and incubated for 10 min at room temperature (RT), and a noninfected and a known MCMV-infected maize leaf crude extract were used as the negative and positive controls, respectively. Then, the membranes were blocked with PBST (a PBS solution containing 0.05% Tween-20) containing 5% skimmed milk powder for 30 min, followed by 1 h of incubation in a diluted anti-MCMV mAb solution at RT. After washing three times with PBST, alkaline phosphatase (AP)-conjugated goat anti-mouse immunoglobulin G (IgG) diluted in PBS containing 5% skimmed milk powder was added and incubated for another 1 h at RT. Afterward, the color reaction was created by adding a combination of NBT/BCIP (nitroblue tetrazolium chloride and 5-bromo-4-chloro-3-indolyl phosphate) substrate solutions for 15 min at RT.

### 2.5. Preparation of AuNP-mAb Conjugate

In this assay, AuNP solution was synthesized using trisodium citrate to reduce HAuCl_4_·3H_2_O as described [41,42,43]. Briefly, 1 mL of 1% HAuCl_4_ solution was added to 96 mL of ultrapure water and heated to boiling under vigorous stirring. Then, 1% trisodium citrate (3 mL) was added immediately to the mixture above and kept boiling for 10 min until the color changed to red. After stirring for another 15 min, the AuNPs, 30 nm in diameter, were successfully prepared for subsequent AuNP-mAb conjugate preparation.

The AuNP-mAb conjugate was prepared via simple electrostatic adsorption. In detail, the pH of the AuNPs solution was adjusted with 0.2 M K_2_CO_3_. Then, 200 μg of anti-MCMV mAb 17C4 was diluted and slowly added to 20 mL of AuNPs solution when stirred. After reacting for 1 h at RT, 10% bovine serum albumin (BSA) was added to block unreacted binding sites. Then, the precipitate was separated by centrifugation for 20 min at 12,000 rpm and resuspended in 2000 μL of PBS buffer (pH 7.4) containing 3% sucrose, 3% BSA, and 0.05% NaN_3_.

### 2.6. Fabrication and Principle of the AuNP-ICTS

**Figure 1:**
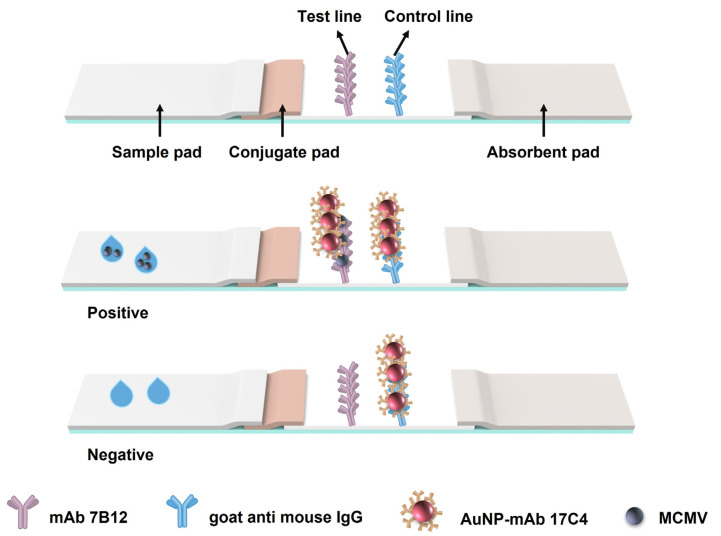
Schematic diagram of AuNP-ICTS for the detection of MCMV.

The ICTS was comprised of the sample pad, conjugate pad, nitrocellulose membrane (Sartorius AG. Millipore, Billerica, MA, USA), and absorbent pad as described previously (Figure 1) [44]. In brief, the sample pad made of glass fibers was pretreated with blocking buffer (PBS containing 3% BSA, pH 7.4) and dried at 37 °C for 5 h. The anti-MCMV-mAb 7B12 (2 mg/mL) and goat anti-mouse IgG (1 mg/mL) were sprayed onto the nitrocellulose membrane at the test (T) line (0.5 μL/cm) and the control (C) line (0.5 μL/cm), respectively. Afterward, all of the above components were assembled orderly onto polyvinyl chloride (PVC) backing with a 2 mm overlap between each of the two adjacent parts (Figure 1). Finally, the strips were cut into 3 mm-wide strips and stored in a desiccated container for further use.

The development of AuNP-ICTS was based on a double-antibody sandwich-format immunoassay. When the analyte of interest, MCMV, was present, it bound with the immune probe AuNP-mAb 17C4 and formed the immunocomplex AuNP-mAb 17C4-MCMV. The resulting immunocomplex moved forward, and its MCMV was captured by immobilized mAb 7B12 on the T line to form a red line, while the free immune AuNP-mAb 17C4 was captured by goat anti-mouse IgG on the C line to form another red line (Figure 1). Thus, the detection result can be observed directly with the naked eye. For negative samples, AuNP-mAb would flow across the T line with no colorimetric signal and be only captured on the C line by goat anti-mouse IgG (Figure 1).

### 2.7. Procedure and Characteristics of the AuNP-ICTS for MCMV Detection

To investigate the sensitivity of the AuNP-ICTS, 60 μL~100 μL of a series of diluted leaf crude extracts from MCMV-infected maize leaves was dropped into the sample pad and reacted for 5 min at RT until the liquid flow nearly stopped. Meanwhile, PBS and crude extract from uninfected maize leaves were used as the blank and negative controls, respectively.

In addition, analytic specificity is another key characteristic of ICTS in practical sensing applications. Therefore, this AuNP-ICTS method was investigated by testing five different plant viruses, including SCMV, southern rice black-streaked dwarf virus (SRBSDV), rice black-streaked dwarf virus (RBSDV), tobacco mosaic virus (TMV), and cucumber mosaic virus (CMV). MCMV- and SCMV-co-infected maize were also tested.

### 2.8. Actual Sample Detection by Dot-ELISA, AuNP-ICTS, and RT-PCR

In order to evaluate the accuracy and effectiveness of the Dot-ELISA and AuNP-ICTS for MCMV detection in actual samples, a total of 20 blinded maize plant samples were analyzed by the two serological assays in this study. At the same time, the monitoring results of the Dot-ELISA and AuNP-ICTS were validated by conventional RT-PCR. For RT-PCR, total RNA was first extracted from each sample (100 mg/each sample) using Trizol reagent (Invitrogen, Carlsbad, CA, USA), and then reverse transcribed with the gene-specific reverse primer, i.e., MCMV-R (5′-ATGGCTCGTGATAAACGGCA-3′), by the HiScript Reverse Transcriptase kit (Vazyme, Nanjing, China). The presence of MCMV in maize samples was then determined using RT-PCR with a set of conserved primers, MCMV-F (5′-GCTCTCGTGAAACACGGACT-3′) and MCMV-R (5′-ATGGCTCGTGATAAACGGCA-3′). Each PCR reaction contained 1 μL of each primer (10 μmol/L), 1 μL of cDNA, 10 μL of 2× Green Taq mix (Vazyme), and 7 μL of sterile deionized water. The reaction cycle was performed as follows: 95 °C for 3 min; 30 cycles of 95 °C for 30 s; 58 °C for 15 s; and 72 °C for 30 s. The final extension was 10 min at 72 °C.

## 3. Results

### 3.1. Preparations of mAbs against MCMV

**Figure 2:**
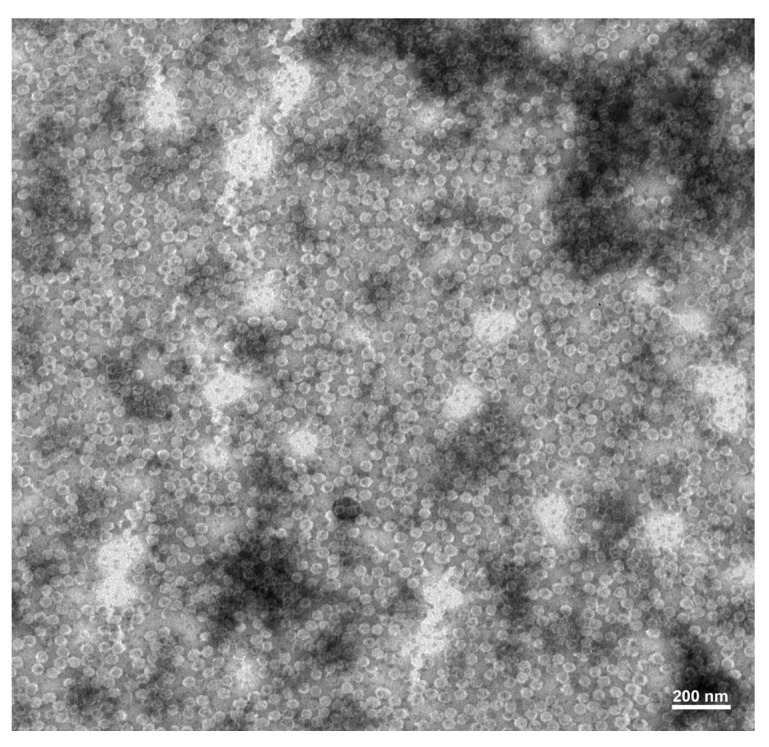
Micrograph of purified MCMV virions by transmission electron microscopy.

To produce anti-MCMV mAbs, purified MCMV particles used as the immunogen were obtained by a differential centrifugation method. According to TEM, the virions showed spherical particles with a mean size of 30 nm (Figure 2). Six hybridoma lines named 7FB, 14G9, 7B12, 17C4, 7C11, and 16D12 secreting anti-MCMV mAbs were obtained via cell fusion, hybridoma cell screening, antibody detection, and cell cloning. These six hybridoma cell lines were separately injected intraperitoneally into pristine-primed BALB/c mice to produce ascites of mAbs. The properties of these resulting mAbs against MCMV are presented in Table 1. All of the mAbs were identified as mouse isotype IgG1, κ light chain, except mAb 16D12, which was determined to be IgG2b, κ light chain. In addition, the IgG yields of six mAbs in ascites ranged from 2.30 to 7.18 mg per milliliter, respectively.

### 3.2. Characterization of Six mAbs and MCMV Detection Using Dot-ELISA

**Figure 3:**
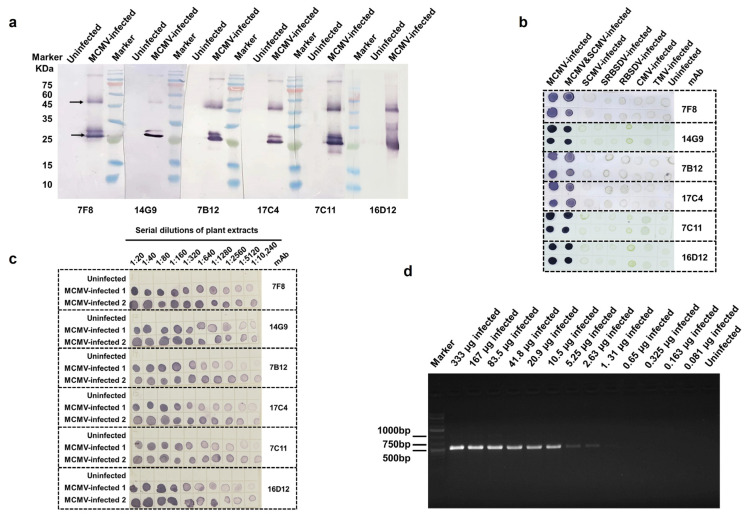
Characterization of six anti-MCMV mAbs. Specificity analyses of six mAbs through Western blot assays (**a**) and Dot-ELISAs (**b**). Sensitivity analyses of Dot-ELISA-based mAbs (**c**) and RT-PCR (**d**). The MCMV-infected sample 2 in Figure 3c was used for RT-PCR detection in Figure 3d.

In order to determine the specificity of the six anti-MCMV mAbs, a Western blot assay was first performed. All of these mAbs reacted specifically with MCMV-infected maize leaf tissues and produced two protein bands at 52 KDa and 26 KDa on the blot membranes, while no such protein bands were found in uninfected maize leaf tissues used as the negative control (Figure 3a). According to the molecular weight of the reacted protein, it is suggested that these mAbs can specifically identify the MCMV CP subunit with 26 KDa, and the band with 52 KDa might be a dimer of the MCMV CP subunit. Moreover, a Dot-ELISA was carried out to further confirm their specificity. All six mAbs can specifically identify MCMV in MCMV-infected and MCMV and SCMV co-infected maize leaf tissues, and did not cross-react with the uninfected maize plant tissues or the other five tested plant viruses, SRBSDV, RBSDV, SCMV, TMV, and CMV (Figure 3b). Therefore, the results above strongly proved that these six mAbs were highly specific to MCMV.

To determine the detection sensitivity of the mAbs and the created Dot-ELISAs, two crude extracts from two MCMV-infected maize leaf samples (MCMV-infected samples 1 and 2) and a crude extract from an uninfected maize leaf tissue were serially two-fold diluted, and each dilution was tested for the presence of MCMV through Dot-ELISA. The results showed that all six Dot-ELISAs separately based on six mAbs (7F8, 7B12, 7C11, 4G9, 16D12, and 17C4) could detect MCMV in 1:10,240 (*w*/*v*, g/mL) diluted maize leaf crude extracts (Figure 3c), equivalent to 0.20 μg of MCMV-infected leaf tissues (Figure 3c). At the same time, conventional RT-PCR was also performed to monitor virus in the MCMV-infected sample 2 in Figure 3c, and the detection endpoint of RT-PCR was determined to be 2.63 μg of infected maize leaf tissues (Figure 3d). Compared with conventional RT-PCR, intriguingly, the Dot-ELISAs based on these six anti-MCMV mAbs showed higher sensitivity, and their detection endpoints are 12.1 times higher than that of RT-PCR. Overall, all six mAbs against MCMV have shown excellent specificity and super-sensitivity and can be used for the sensitive and correct detection of MCMV in actual samples.

### 3.3. Performance of AuNP-ICTS for MCMV Detection

**Figure 4:**
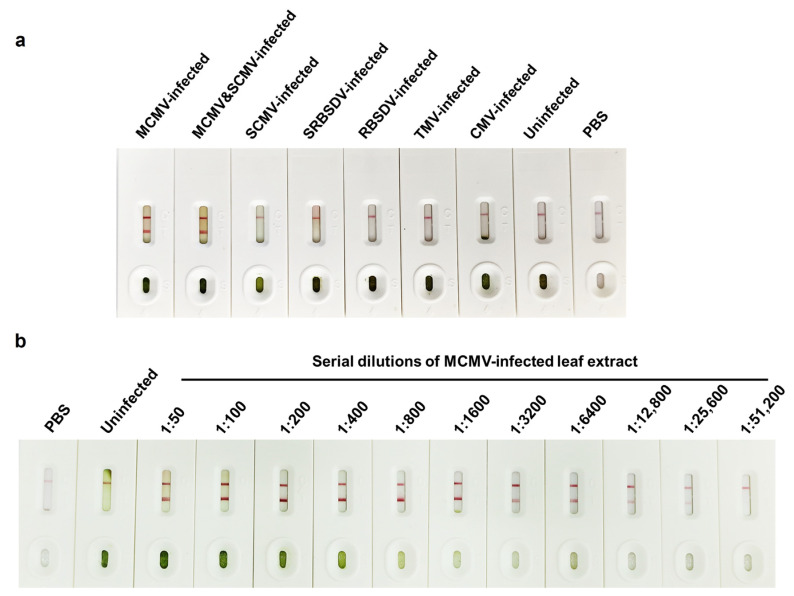
Characteristics of the newly developed AuNP-ICTS for MCMV detection. (**a**) Specificity analysis result of the AuNP-ICTS using five different plant viruses and uninfected maize plants as the samples. (**b**) Sensitivity analysis result of the AuNP-ICTS using serially diluted MCMV-infected maize leaf crude extracts as the monitoring samples.

ELISA-based methods often require approximately 3~5 h to be performed. In this work, a rapid, simple, and user-friendly AuNP-ICTS was first established for monitoring MCMV in maize plant samples. The specificity of AuNP-ICTS was determined using five different plant viruses, including SCMV, SRBSDV, RBSDV, TMV, and CMV, and uninfected maize plants. When MCMV-infected or MCMV and SCMV-co-infected maize samples were added to the sample pads, two red lines were seen on both the C and T lines in 5 min (Figure 4a). For the other five plant viruses and uninfected maize plants, we observed only the colorimetric signal on the C line, while there was no red AuNPs deposition on the T line. These results above indicated that the newly created AuNP-ICTS has excellent specificity for MCMV monitoring. As the colorimetric results show in Figure 4b, the red color on the T lines gradually weakens with the increase of the dilution ratio of the MCMV-infected maize leaf crude extract. The detection endpoint of the established AuNP-ICTS for the infected maize leaf crude extracts was determined to be a 1:25,600-fold dilution (*w*/*v*, g/mL), which amounted to 2.34 μg MCMV-infected plant tissues, while the detection endpoint of the conventional RT-PCR is 2.63 μg MCMV-infected plant tissues (Figure 3d), indicating that AuNP-ICTS is more sensitive than RT-PCR. Furthermore, the detection process, including sample treatment for AuNP-ICTS, can be completed within 10 min without any equipment. Thus, a super-sensitive, specific, and rapid AuNP-ICTS was successfully created and can easily realize MCMV detection in 10 min.

### 3.4. Detection Results of Actual Samples by Dot-ELISA, AuNP-ICTS, and RT-PCR

**Figure 5:**
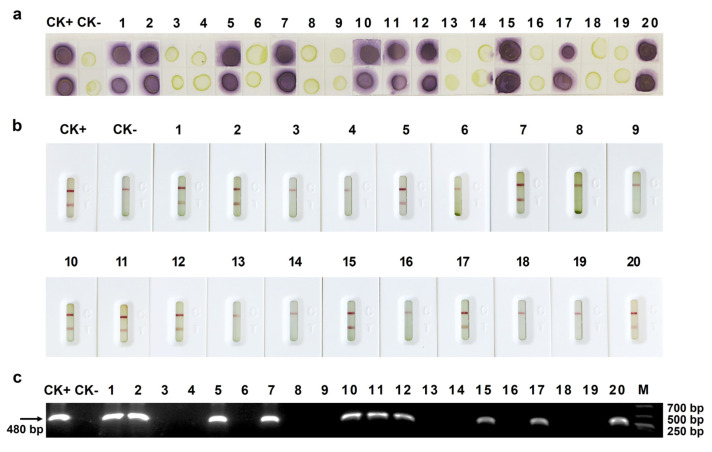
Detection of MCMV infection in maize leaf samples through the newly created Dot-ELISA (**a**), AuNP-ICTS (**b**), and RT-PCR (**c**). The RT-PCR products were about 480-bp gene segment. CK+ and CK- were known MCMV-infected and uninfected maize leaf samples, respectively.

To evaluate the effectiveness and accuracy of the Dot-ELISA and AuNP-ICTS for MCMV monitoring, 20 blinded maize samples collected from maize fields in Yunnan Province, China, intercepted at customs ports, and maize plants inoculated with MCMV in our glasshouse were screened for the presence of MCMV. The results of Dot-ELISA and AuNP-ICTS simultaneously and conformably showed that 10 of 20 samples, including samples 1, 2, 5, 7, 10, 11, 12, 15, 17, and 20, were infected with MCMV, which is in good concordance with the result obtained by RT-PCR using the primer pairs MCMV-F and MCMV-R (Figure 5a–c). Therefore, the newly created Dot-ELISA and AuNP-ICTS exhibited favorable application potential for MCMV detection in plant samples.

## 4. Discussion

With global warming and the international trade in agricultural products increasing, MCMV causes more and more losses in the maize industry worldwide. Thus, it is of great significance to establish specific, sensitive, and rapid detection methods to prevent the spread of MCMV in domestic maize-producing areas and different countries. Effective integrated management measures are dependent on the timely and proper detection of MCMV. Over the past few decades, the detection methods against MCMV have improved from biological detection of indicator plants to serological and molecular biological techniques. RT-PCR has been widely used for the detection of MCMV in East Africa, South America, and Asia [7,19,20,21]. In 2010, Zhang and colleagues established a real-time TaqMan RT-PCR for monitoring MCMV, and its sensitivity was ten-fold higher than conventional RT-PCR [22]. Compared to PCR-based molecular biological methods that require trained technicians and expensive equipment, serological techniques are generally considered the most practical, cost-effective, and reliable methods for plant virus monitoring due to their ease of use, excellent specificity, and high sensitivity [45,46,47,48,49]. High-quality antibodies are the most critical reagents for serological assays and determine the specificity and sensitivity of immunoassays. Polyclonal antibodies (pAbs) and mAbs are commonly used as biorecognition reagents in serological assays for virus monitoring. Due to the pitfall of insufficient specificity of pAbs that recognize multiple epitopes, highly specific and highly sensitive mAbs obtained by artificial screening have been widely used in serological assays for plant virus detection [36,37]. In addition, CPs of all reported MCMV isolates worldwide share more than 95% of their nucleotide identity [3], which is a huge advantage for the development of reliable serological diagnostic techniques. Our group previously produced four murine mAbs against MCMV in 2013 and created TAS-ELISA and Dot-ELISA for monitoring MCMV [17]. However, that work did not develop an ICTS for MCMV detection. ICTSs, as a rapid, easy-to-use, and low-cost detection tool, have been used to monitor diverse plant viruses on site and are now the most widespread tool for virus detection [34,47,50,51,52]. In 2023, Lei and colleagues established a CRISPR/Cas12a-based lateral flow assay for MCMV detection by combining one-step reverse-transcription recombinase-aided amplification [53], but this assay requires a metal incubator and takes 1 h to complete the whole detection procedure, making it unsuitable for on-site testing. 

In this study, we produced six super-sensitive and highly specific mAbs against MCMV and then established newly super-sensitive and specific Dot-ELISAs and AuNP-ICTS to realize visual and super-sensitive detection of MCMV in plant samples. All six obtained mAbs can bind specifically to MCMV CP in infected maize leaf tissues and have no cross-reaction with the other five tested plant viruses or uninfected maize plant tissues. Six Dot-ELISAs using anti-MCMV mAbs as the detection antibody were created, and their detection endpoints were up to 0.20 μg MCMV-infected maize leaf tissues, surprisingly, which is 12.1 times higher than that of the conventional RT-PCR. In addition, rapid and user-friendly AuNP-ICTS based paired mAbs 7B12 and 17C4 were created for on-site testing of MCMV. The created AuNP-ICTS could detect virus in 25,600-fold diluted crude extracts (*w/v*, g/mL) from MCMV-infected maize leaf within 10 min (Figure 4), and its sensitivity was slightly higher than that of RT-PCR. The test results of 20 blind maize samples proved the accuracy of the established Dot-ELISA and AuNP-ICTS tests to monitor MCMV in maize plant samples at the levels of maize fields and ports of entry. Therefore, the newly created Dot-ELISA and AuNP-ICTS have great application potential for the on-site detection of MCMV and provide guidelines for monitoring other plant viruses.

## Figures and Tables

**Table 1:** Properties of mAbs against MCMV.

mAbs	Isotypes	Ascites Titer	IgG Yield in Ascites (mg/mL)
7F8	IgG1, κ	10^−7^ *	7.18
14G9	IgG1, κ	10^−7^	5.36
7B12	IgG1, κ	10^−7^	3.98
17C4	IgG1, κ	10^−7^	5.09
7C11	IgG1, κ	10^−7^	4.74
16D12	IgG2a, κ	10^−7^	2.30

* The ascites titers were determined by an indirect ELISA.

## Data Availability

Not applicable.
